# Effect of positioning and expiratory rib-cage compression on atelectasis in a patient who required prolonged mechanical ventilation: a case report

**DOI:** 10.1186/s13256-022-03389-5

**Published:** 2022-06-23

**Authors:** Takuya Hosoe, Tsuyoshi Tanaka, Honoka Hamasaki, Kotomi Nonoyama

**Affiliations:** Department of Rehabilitation, Nagoya City Midori Municipal Hospital, 1-77, Shiomigaoka, Midori-ku, Nagoya-shi, Aichi 458-0037 Japan

**Keywords:** Prolonged mechanical ventilation, Lung, Pulmonary complications, Atelectasis, Rehabilitation, Expiratory rib-cage compression

## Abstract

**Background:**

Pulmonary complications can be caused by intraoperative mechanical ventilation. In particular, prolonged mechanical ventilation is associated with a high mortality rate, a risk of pulmonary complications, prolonged hospitalization, and an unfavorable discharge destination. Pre- and postoperative rehabilitation are important for the resolution of pulmonary complications in acute cases. However, there has been a lack of studies on interventions for pulmonary rehabilitation of patients with chronic pulmonary complications caused by prolonged mechanical ventilation. Accordingly, we describe the effect of pulmonary rehabilitation in such a patient.

**Case presentation:**

We examined a 63-year-old Japanese woman with hypoxic–ischemic encephalopathy after subarachnoid hemorrhage who required prolonged mechanical ventilation. Radiographic and computed tomographic images revealed atelectasis of the right upper lobe. In addition, this atelectasis reduced the tidal volume, minute volume, and oxygen saturation and caused an absence of breath sounds in the right upper lobe during auscultation. We aimed to ameliorate the patient’s atelectasis and improve her ventilation parameters by using positioning and expiratory rib-cage compression after endotracheal suctioning. Specifically, the patient was seated in Fowler’s position, and mild pressure was applied to the upper thorax during expiration, improving her inspiratory volume. Immediately, breath sounds were audible in the right upper lobe. Furthermore, resolution of the patient’s atelectasis was confirmed with chest radiography performed on the same day. In addition, her ventilation parameters (tidal volume, minute volume, and oxygen saturation) improved.

**Conclusions:**

Our results indicate that physical therapists should consider application of specific positioning and expiratory rib-cage compression in patients who exhibit atelectasis because of prolonged mechanical ventilation.

## Background

Mechanical ventilation increases the risk of ventilator-induced lung injuries such as barotrauma and increased vascular permeability [1]. Pulmonary complications, such as pneumonia and atelectasis, can be caused by intraoperative mechanical ventilation [2, 3]. In particular, prolonged mechanical ventilation (PMV) is associated with a high mortality rate, the occurrence of pulmonary complications, prolonged hospitalization, and an unfavorable discharge destination [4–6]. PMV is defined as the requirement for more than 21 consecutive days of mechanical ventilation for at least 6 h per day [7].

Intraoperative mechanical ventilation and lung, cardiac, or abdominal surgery may lead to postoperative lung complications [2, 3, 8–11], and bed rest causes a decline in muscle strength and respiratory functionality [12]; therefore, pre- and postoperative rehabilitation protocols are important for risk reduction and management of acute pulmonary complications and muscle loss [13, 14]. However, there have been few studies examining interventions for rehabilitation of chronic pulmonary complications caused by PMV. Accordingly, in this case report, we describe the effect of pulmonary rehabilitation on pulmonary complications in a patient who required PMV.

## Case presentation

**Fig. 1:**
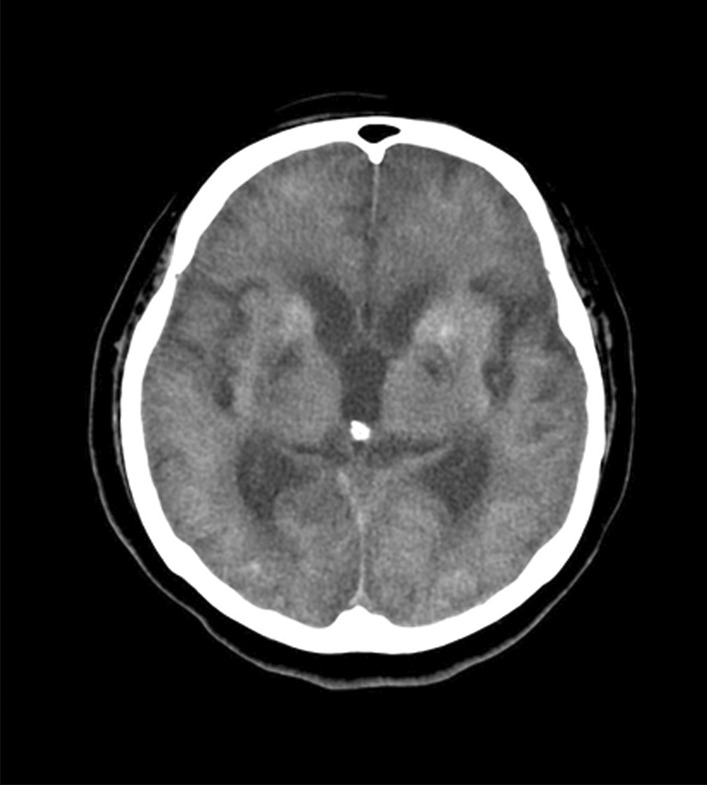
Computed tomography images of the head. Images of the whole brain indicated hypoxic encephalopathy

We examined a 63-year-old Japanese woman with hypoxic–ischemic encephalopathy after subarachnoid hemorrhage who required PMV. She had a medical history of being treated with catheter ablation for atrial fibrillation 1 year before the onset of hypoxic–ischemic encephalopathy after subarachnoid hemorrhage. The patient was married, with a history of one successful pregnancy. Additionally, she had lived inside and outside her home as a housewife after giving birth. She had a medication history of using oral anticoagulants (15 mg/day). There was no history of smoking or alcohol consumption. This patient developed hypoxic encephalopathy because of cardiac arrest lasting more than 10 min after subarachnoid hemorrhage (Fig. 1). In the intensive care unit of the previous hospital, mechanical ventilation and rehabilitation were initiated as spontaneous respiration had stopped after the onset of the disease. Rehabilitation was performed at the previous hospital until 4 months after the onset of the disease, but the patient did not start to breathe spontaneously. Thus, at that point, the patient was transferred to our hospital for further rehabilitation.

**Fig. 2:**
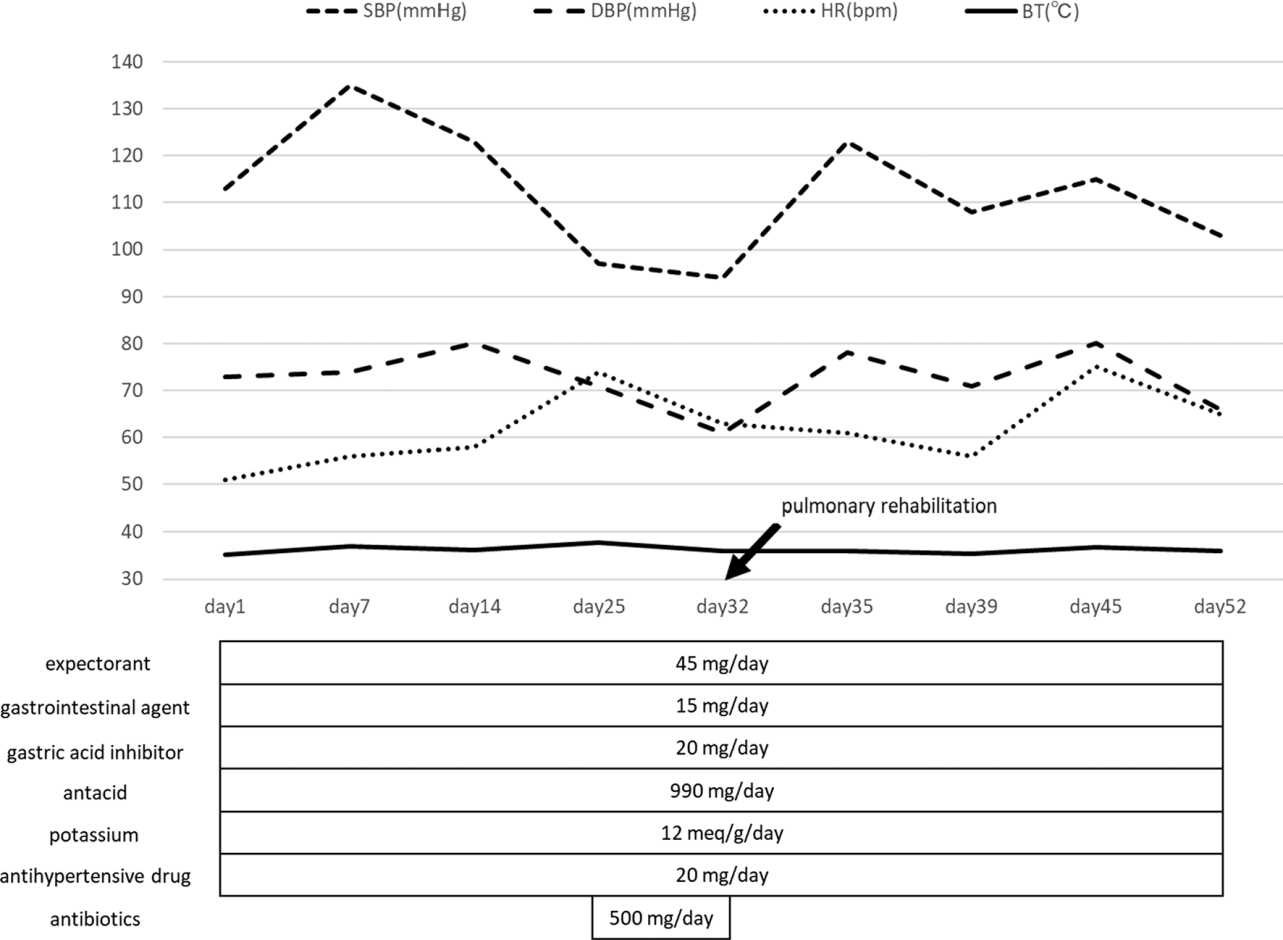
Vital signs and medications at 1 month

On admission, physical and neurological examinations showed that the level of consciousness was E4V1M4 on the Glasgow Coma Scale (GCS), making communication difficult. No significant limitation was found in the range of motion of the neck, limbs, and trunk. However, the deep tendon reflexes of the limbs and pathological reflexes were hypoactive or absent, and little spontaneous movement of the limbs was observed. Spontaneous breathing was also absent, as noted in the previous hospital. Vital signs (blood pressure, heart rate, and body temperature) at time of admission and during hospitalization were variable due to hypoxic–ischemic encephalopathy. Medications, including expectorant, gastrointestinal agent, gastric acid inhibitor, antacid, potassium, antihypertensive drugs, and antibiotics, were administered via a nasogastric tube (Fig. 2). The results of laboratory findings at the time of admission to our hospital were as follows: complete blood count (WBC = 6300/μL, RBC = 3,910,000/μL, Hb = 12.2 g/dL, PLT = 273,000/μL), liver function (AST = 18 U/L, ALT = 29 U/L, T-Bil = 0.4 mg/dL), renal function (BUN = 27.3 mg/dL, Cre = 0.73 mg/dL), urinalysis (opacity = 1+, PH = 8.5), and inflammatory response (CRP = 0.39 mg/dL). Serology was negative for hepatitis B and C; however, microbial culture test revealed *Pseudomonas aeruginosa* in sputum and urine.

**Fig. 3:**
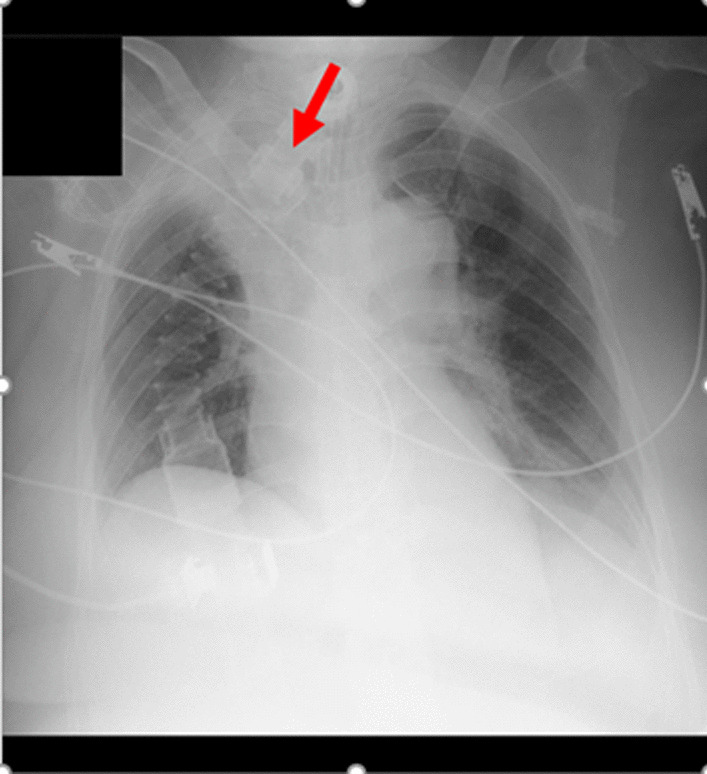
Chest radiograph. Red arrow indicates presence of atelectasis

**Fig. 4:**
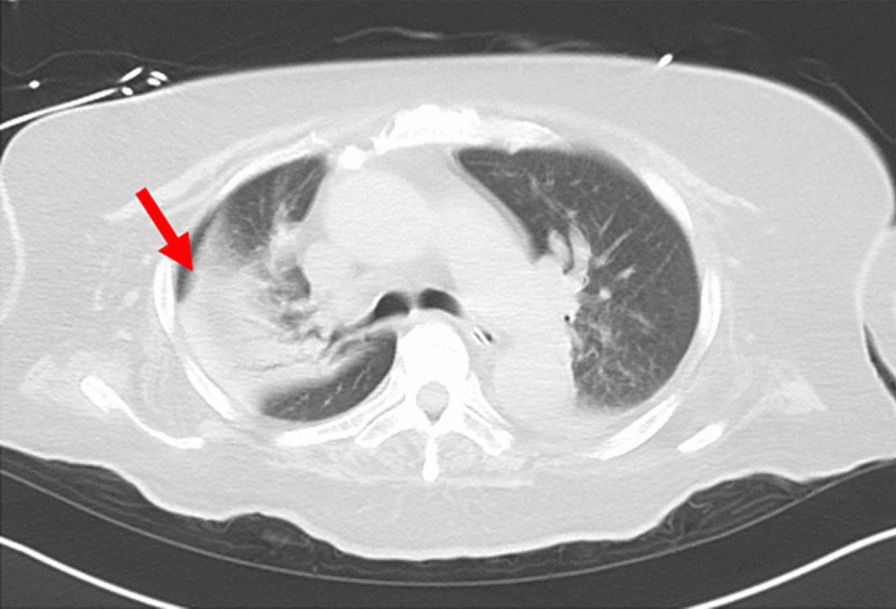
Computed tomography images of the chest. Red arrow indicates presence of atelectasis

**Fig. 5:**
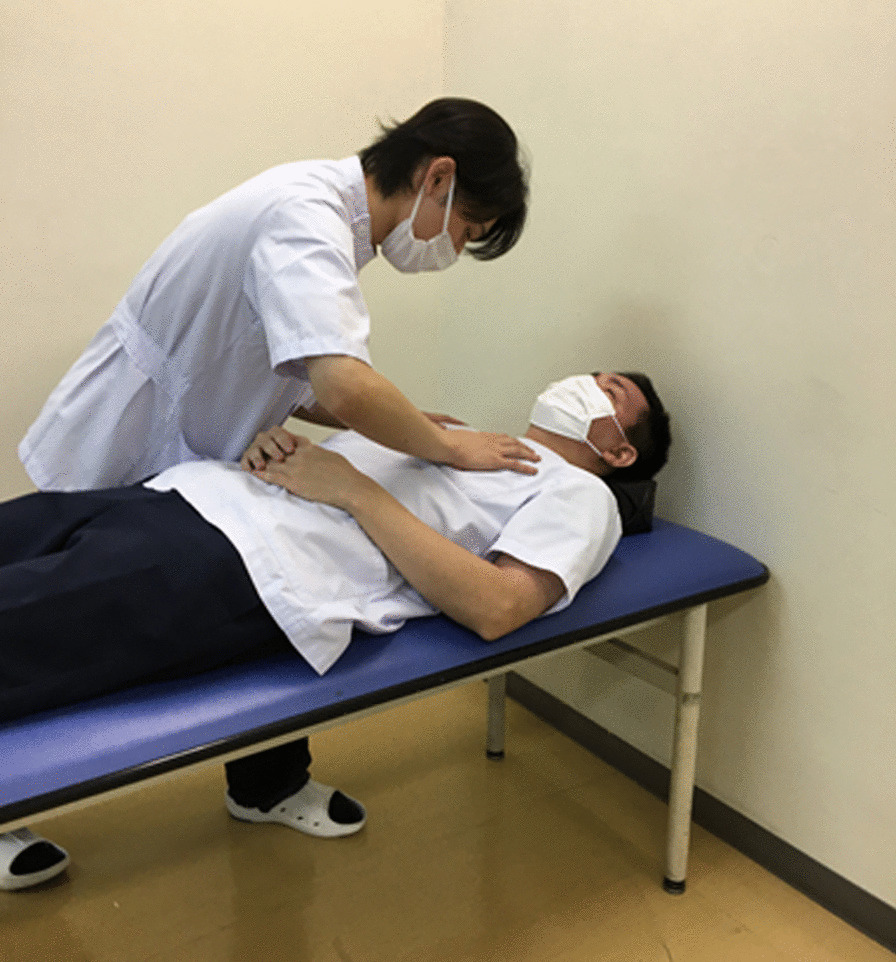
Expiratory rib-cage compression. The technique consisted of application of mild pressure to the upper thorax during expiration, with the aim of increasing the inspiratory volume of the upper lobe

The Trilogy 100 plus ventilator (Koninklijke Philips N.V., Amsterdam, Netherlands) was used for ventilatory management at our hospital, and the pressure-controlled mode was adopted, similar to the management during the patient’s previous hospitalization (Table 1). The patient’s breath sounds were clear except for coarse crackles owing to accumulation of pulmonary secretions. Pulmonary complications were not observed upon chest radiography immediately after admission to our hospital. However, 1 month after admission to our hospital, atelectasis of the right upper lobe was observed for three consecutive days upon radiography (Fig. 3) and computed tomography (Fig. 4). This, in turn, reduced the tidal volume, minute volume, and oxygen saturation, and caused an absence of breath sounds in the right upper lobe during auscultation. We aimed to ameliorate the patient’s atelectasis and improve her ventilation parameters with positioning [15] and expiratory rib-cage compression (Fig. 5) for pulmonary rehabilitation after endotracheal suctioning. The patient was seated in Fowler’s position, and the expiratory rib-cage compression involved the application of mild pressure to the upper thorax during expiration, which tends to increase the inspiratory volume of the right upper lobe. Endotracheal suctioning was performed according to American Association for Respiratory Care clinical practice guidelines [16].

**Table 1:** Ventilation parameters used in this case

Ventilation parameter	Value
Mode of ventilation	PC
IPAP (cmH_2_O)	19
EPAP (cmH_2_O)	8
Inspiratory time (seconds)	1.2
Rise time (seconds)	1.0
Flow trigger sensitivity (l/minute)	3
FiO_2_ (%)	25

PC, pressure-controlled; IPAP, inspiratory positive airway pressure; EPAP, expiratory positive airway pressure; FiO_2_, fraction of inspired oxygen

**Fig. 6:**
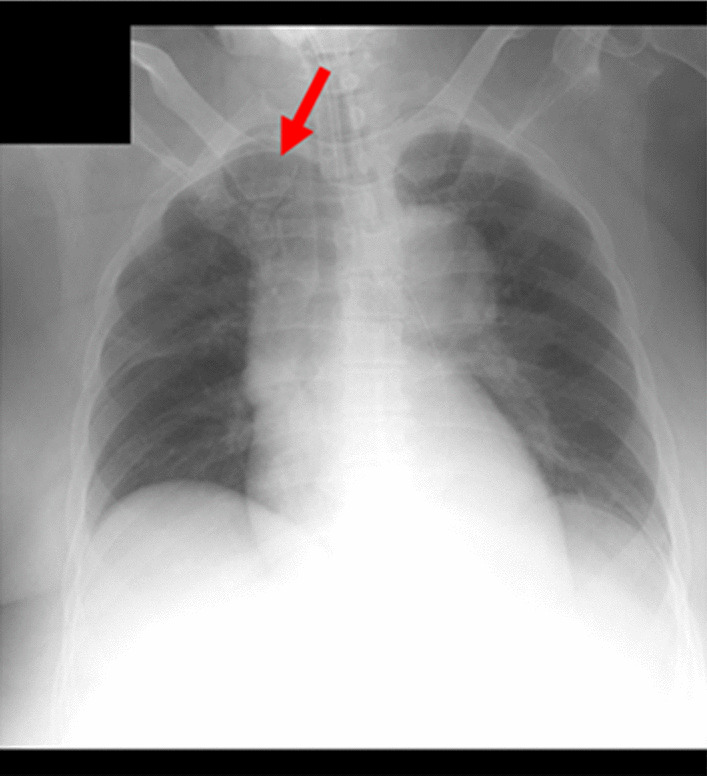
Chest radiograph after pulmonary rehabilitation. Red arrow indicates amelioration of atelectasis

Breath sounds were audible in the patient’s right upper lobe as soon as the intervention started. Furthermore, amelioration of her atelectasis was observed upon chest radiography performed on the same day (Fig. 6). In addition, her ventilation parameters (tidal volume, minute volume, and oxygen saturation) improved (Table 2).

**Table 2:** Comparison between pre- and postintervention respiratory parameters

Respiratory parameter	Preintervention	Postintervention
Breath sounds	Absent	Normal
Tidal volume (ml)	286	419
Minute volume (l/minute)	4.1	5.2
End-tidal CO_2_ (mmHg)	40	37
Oxygen saturation (%)	88	97
Maximum inspiratory flow (l/minute)	27.2	36.8

**Fig. 7:**
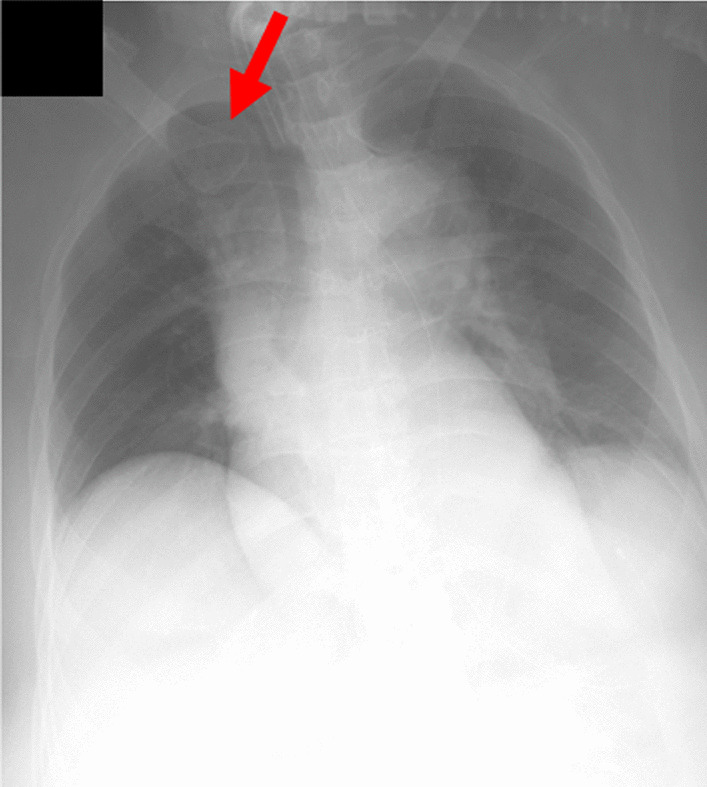
Chest radiograph after 6 months of pulmonary rehabilitation. Red arrow indicates amelioration of atelectasis

Physical and neurological examinations at 6 months after pulmonary rehabilitation revealed a Glasgow Coma Scale (GCS) score of E4V1M4, no significant limitation on range of motion, hypoactive or absent deep tendon and pathological reflexes, and absence of spontaneous breathing. The vital signs were as follows: SBP, 119 mmHg; DBP, 83 mmHg; HR, 57 bpm; and BT, 36.1 °C. The results of laboratory tests included CBC (WBC = 8600/μL, RBC = 3,920,000/μL, Hb = 12.4 g/dL, PLT = 238,000/μL), liver function (AST = 10 U/L, ALT = 26 U/L, T-Bil = 0.4 mg/dL), and renal function (BUN = 25.9 mg/dL, Cre = 0.81 mg/dL). Chest radiographs showed improvement in atelectasis in the right upper lobe after pulmonary rehabilitation (Fig. 7).

## Discussion and conclusions

The current patient with hypoxic–ischemic encephalopathy after subarachnoid hemorrhage had been on long-term mechanical ventilation since the onset and presented with atelectasis 5 months after onset. We thus aimed to improve the condition using positioning and respiratory assistance. In acute situations, pre/postoperative respiratory rehabilitation of patients undergoing lung or cardiac surgery reportedly prevents development of pulmonary complications [17, 18].

However, there have been only a few studies on the effects of respiratory rehabilitation on chronic pulmonary complications. The number of deaths occurring at 30 and 90 days after lung or cardiac surgery is higher in patients with pulmonary complications than in those without [2, 8, 9]; therefore, in daily practice, there is a need for treatment optimization for patients with pulmonary complications. However, there is a need to consider application of pulmonary rehabilitation in chronic cases, such as for patients requiring PMV. In our patient, positioning and expiratory rib-cage compression were associated with an increase in specific lung volume, which led to immediate resolution of the atelectasis.

Expiratory rib-cage compression increases tidal volume and secretion clearance [19, 20]; it involves the application of mild pressure on the upper or lower thorax, which increases the expiratory volume of the lungs in a specific area. This method is actively promoted for respiratory rehabilitation and is one of the most practiced interventional methods in Japan. In addition, as expiratory rib-cage compression is very simple to perform and does not require special equipment, it is easy to incorporate into rehabilitation protocols.

Manual hyperinflation reportedly improves lung compliance. It is associated with short-term improvements in lung compliance, oxygenation, and secretion clearance [21]. However, manual hyperinflation has also been associated with acute lung injury [22]. A benefit of expiratory rib-cage compression compared with manual hyperinflation is that no special equipment is needed for performing it. Moreover, as expiratory rib-cage compression is performed by placing only mild pressure on the upper thorax during expiration, the risk of lung injury, such as barotrauma, is considered low. However, there is a lack of studies regarding the effects of expiratory rib-cage compression. Therefore, further research is needed for consideration of its risks and benefits in patients with chronic lung injuries.

In conclusion, in our patient, who required PMV, the resulting atelectasis improved immediately after application of specific positioning and expiratory rib-cage compression. Hence, physical therapists should consider this treatment for patients exhibiting atelectasis due to PMV.

## Data Availability

The datasets generated and/or analyzed during the current study are not publicly available because of patient privacy but are available from the corresponding author on reasonable request.

## Abbreviation

PMV: Prolonged mechanical ventilation
